# Differential proteomic alterations between localised and metastatic prostate cancer

**DOI:** 10.1038/sj.bjc.6603274

**Published:** 2006-08-01

**Authors:** B S Taylor, S Varambally, A M Chinnaiyan

**Affiliations:** 1 Department of Pathology, University of Michigan Medical School, Ann Arbor, MI 48109, USA; 2 Bioinformatics Program, University of Michigan Medical School, Ann Arbor, MI 48109, USA; 3 Comprehensive Cancer Center, University of Michigan Medical School, Ann Arbor, MI 48109, USA; 4 Department of Urology, University of Michigan Medical School, Ann Arbor, MI 48109, USA

**Keywords:** metastasis, cancer proteomics, bioinformatics, systems biology, prostate cancer, tumour marker

## Abstract

Molecular alterations in the prostate cancer proteome mediate the functional and phenotypic transformation from clinically localised to metastatic cancer, a transition that drives patient's mortality and challenges therapeutic intervention. A first approximation of differential proteomic alterations stratified by disease stage has yielded repertoires of potential diagnostic and prognostic markers, multiplex signatures of predictive value, and yield fundamental insight into molecular commonalities in cancer progression. Deciphering these causative proteomic alterations from the molecular noise will continue to mature our understanding of tumour biology and drive new computational and integrative approaches to model a system's view that accommodates the heterogeneity of prostate cancer progression.

Although clinically localised prostate cancer can be treated with androgen ablation, surgical resection or radiation, its transition to metastatic disease is almost uniformly fatal. Understanding the molecular hallmarks of this transition has been the target of extensive study by the wider research community. In this context, a multitude of both diagnostic and prognostic tissue and serum biomarkers have been proffered as viable supplements to the standard clinical parameters in use now. These include prostate-specific antigen, Gleason score and clinical stage; however, these are individually opaque to sensitive disease fate decisions. In parallel, there are emerging efforts in integrative and systems approaches to prostate cancer progression. These promise not only molecular profiles and signatures, but also models of progression that assimilate the heterogeneity and complexity of a decidedly continuous and nonlinear biological process. We focus here on recent approaches and discoveries, and specifically in the context of those proteomic alterations between the later, progressive stages of prostate cancer.

## DISCOVERY AND DIVERSITY OF ALTERATION TYPES AND A VECTOR OF PROTEOMIC CHANGE

The explosion of high-throughput platforms for protein-level analysis, primarily driven by mass spectrometry and array-based methodologies, represents a transition to an exponential phase in oncoproteomics. The biomedical literature is replete with prostate cancer profiling efforts and biomarker discovery in serum, urine, and a growing number in tissue along with variations on early detection platforms and technologies (Wulfkuhle *et al*, 2003). Many of these are exploiting the more immediate translational opportunities in clinical proteomics. These include biofluid profiling, despite the challenges of reproducibility from physiological variability. As our focus here is on proteomic alterations in the prostate cancer tissue proteome, we benefit from circumventing the many problems of secondary and tertiary clinical conditions that drive this physiological variation between and among patient cohorts. Alternatives exist to these explicit and discrete protein biomarker identification techniques. One such method is SELDI-TOF-derived proteomic pattern diagnostics coupled to both significant bioinformatics and up- and downstream sample processing. Nevertheless, much of the existing work across all sample classes confirms a confounding paradox in the cancer proteomics community. Although the vast majority of FDA-approved biomarkers are indeed proteins, none were discovered via high-throughput experimentation and few are used in routine clinical practice (Ludwig and Weinstein, 2005). Consequently, work of our group and others focuses on satisfying the twin and overlapping goals of viable biomarker discovery and deconvoluting the proteomic mechanisms in prostate cancer progression.

During the stages of cancer progression, the tissue proteome fluctuates exquisitely reflecting a dynamism fundamental to the complexity of the process. It is a constantly moving target, the combination of changes that span a vast array of both physical and functional modifications, as well as proteins being secreted and culled from the circulating proteome or degraded by deregulated proteolytic processes. Modifications include differential expression and protein coexpression, most frequently quantified in clinical samples with chemically incorporated stable isotope labelling coupled to high mass accuracy mass spectrometry. Another is altered localisation characterised by the direct analysis of tissue sections with methods such as MALDI imaging mass spectrometry (IMS) (Caprioli, 2005). Other changes include modifications to networks of protein–protein interactions mediating nearly all of the molecular events in disease progression, which are amenable to protein microarray analysis. Additionally, and playing a pivotal functional role in oncogenesis and progression, are post-translational modifications, which require platforms of high mass accuracy and increasing levels of sensitivity for high-throughput discovery. Finally, it is worth mentioning the effect that isolation and extraction techniques, such as laser-capture microdissection (LCM), are having on the known altered proteome in prostate tissue, whether clinically localised or metastatic disease. Althoughly this method aids immeasurably in generating homogenous tumour cell populations for study and certainly improves the specificity of electrophoretic methods like 2D-PAGE in biomarker discovery, it may also help unmask critical alterations otherwise unavailable at the whole-tissue level. The tumour microenvironment is a heterogeneous mix of cell types that includes stromal constituents. In lieu of pure epithelial cell models, LCM in combination with better computational tools is helping to sift out functionally contributing stromal proteins from those that are truly contamination. This will capture a more complete picture of the proteomic interplay in the tumour microenvironment that includes potentially important stromal components. Together, these technologies are generating individual and combinations of proteomic targets.

Although rigid protein repertoires generated from these platforms are informative, they are single dimensional. As a result, their sufficiency is subordinate to otherwise factorial molecular behaviour. Much of the newest proteomic profiling work is generating steady-state first approximations of the differential proteome between discrete stages of prostate cancer (Varambally *et al*, 2005). Furthermore, this work represents an analytical shift towards integrative molecular study, a systems approach to dissecting the molecular events that dictate prostate cancer progression. In this context, there are significant qualitative changes happening both locally and globally at the proteomic level, independent of any single protein, which might be thought of as gross proteomic remodelling between disease stages.

Our group and others have observed, via high-throughput tissue microarray analysis, a general vector of qualitative change at the protein level between clinically localised and metastatic tissue extracts. Leveraging immunohistochemically compatible antibodies, and aside from differences in individual alterations, we are witnessing broader trends in epithelial cell staining paralleling discrete transitions in progressive disease (Varambally *et al*, 2005). This includes metastatic tumours showing reduced membranous staining as compared to localised tumours, which might be predicted by canonical models of adhesion, invasion and proteolytic degradation. Further, these may become increasingly accessible to characterisation as methods for mass spectrometry-based membrane proteomics improve (Wu and Yates, 2003). Additionally, cellular conflation and tumour composition also affect proteomic behaviour, which is uncovered in subsequent staining. This is attributable to factors including the increase in density of tumour cells potentiating higher protein expression levels in localised and metastatic prostate cancer, as well as shifts in stromal to epithelial components between disease stages. Tumours also demonstrate variable levels of subcellular and, especially, nuclear protein expression across a range of markers, which may again be a product of cellular density and not necessarily a direct product of tumour progression. These higher-level observations purport a holistic approach to tissue proteome analysis. Vertically integrating between the depths of analysis in individual protein alterations to the higher-level breadth of gross behavioural observations allows for larger and more comprehensive models of the prostate cancer tissue proteome.

## ALTERATIONS IN A GLOBAL PROGRESSION SIGNATURE

There is currently a relative paucity of work on profiling proteomic alterations in tissues between the various disease stages in prostate cancer. This is more often dealt with at the transcriptomic level (Stanbrough *et al*, 2006), from which the vast majority of existing prostate cancer markers, prognostic or otherwise, have been characterised. However, the newest parallel efforts in systematic protein analysis are further elucidating the behaviour of these and many more, of which we have sampled a small fraction (Table 1). A portion of these is extensively well-studied and fundamentally archetypal of high-throughput profiling for disease markers and are well-reviewed elsewhere (Kumar-Sinha and Chinnaiyan, 2003). Nevertheless, we spend some time here discussing a subset of these; a transcriptional repressor, enzyme, kinase and two nuclear proteins, which serve as an example of the multi-dimensionality of proteomic alterations between stages of prostate cancer. Additionally, that all of these were discovered or recapitulated by our laboratory's recent work with a single high-throughput immunoblot approach emphasises the capacity of new experimental and integrative approaches to model large swaths of the differential prostate cancer proteome (Varambally *et al*, 2005).

The first of these proteins is EZH2, the human homologue of the *Drosophila* protein Enhancer of Zeste (E(Z)), a member of the Polycomb group of proteins. It is involved in epigenetic gene silencing. Recently, it has been linked via DNA methyltransferases, suggesting a direct connection between two key epigenetic repression systems (Vire *et al*, 2006). Our group evaluated the expression of EZH2 by immunohistochemistry using tissue microarrays from patients with either clinically localised or hormone-refractory prostate cancer. The result indicated poorer prognosis with increased EZH2-positive staining and suggests prostate cancer progression and metastasis with increased EZH2 expression (Varambally *et al*, 2002). In addition to prostate cancer, we have demonstrated that EZH2 protein levels were strongly correlated with breast cancer aggressiveness and promote neoplastic transformation of breast epithelial cells (Kleer *et al*, 2003). We have also shown that EZH2 promotes anchorage-independent growth and cell invasion and endows primary cells with a proliferative advantage. Additionally, its gene locus is specifically amplified in several primary tumours (Sudo *et al*, 2005; Bachmann *et al*, 2006). Importantly, studies have demonstrated the intrinsic enzymatic activity of EZH2 as a histone H3 lysine 27 methyltransferase, which is critical to its function as a transcriptional repressor and oncogene (Cao and Zhang, 2004). Thus, if a small molecule inhibitor can be identified against EZH2 enzymatic activity or its protein–protein interactions, this may have utility as a viable therapeutic against tumours expressing high levels of EZH2. Based on the early success of HDAC inhibitors, we expect that targeting EZH2 may be a more specific and rational therapy.

**Figure 1:**
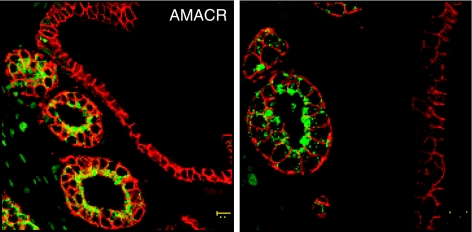
Prostate cancer tissue staining for AMACR. High levels of the prostate cancer biomarker AMACR (green) stained mainly in clinically localised prostate cancer glands compared with internal adjacent benign glands. A higher magnification image is included on the right, and membrane E-cadherin expression is shown in red.

Another protein, alpha-methylacyl-CoA racemase (AMACR), is a peroxisomal and mitochondrial enzyme involved in the *β*-oxidation of branched fatty acids. It was discovered to have specifically increased expression in prostate cancer epithelia (Rubin *et al*, 2002). Our studies have also demonstrated that AMACR enzymatic activity is increased in prostate cancer relative to benign epithelia (Figure 1) (Kumar-Sinha *et al*, 2004). Of particular interest, we have identified a humoral immune response against this enzyme in prostate cancer patient serum (Sreekumar *et al*, 2004). Furthermore, we have shown that AMACR expression is highest in localised prostate cancer and decreases in metastatic disease, and subsequent reductions in AMACR expression in the former is associated with an increased rate of biochemical recurrence (Rubin *et al*, 2005). Although this reduction in hormone-refractory metastasis has been repeatedly observed, little correlation between it and other clinical parameters exists in prostate cancer progression. Additionally, its independence of androgen receptor-mediated signalling has spawned associations between AMACR protein expression loss and tumour dedifferentiation, and the latter's appropriation of pathway control to maintain the formers enzymatic and energy regulation activity. Aside from these observations, a causal link between AMACR protein expression patterns and prostate carcinogenesis is yet to be elucidated.

On the other hand, the centrosome-associated oncogenic kinase Aurora-A, or STK15, belongs to a family of serine/threonine kinases. It has been implicated in playing a crucial role in the control of mitosis. Overexpression of STK15 results in chromosomal aberration, genomic instability and tumorigenesis. This kinase is also known to be amplified in a number of human cancers and tumour cell lines (Jeng *et al*, 2004). STK15 was also shown to regulate the p53 pathway by inducing increased degradation of p53, leading to aberrant checkpoint responses and facilitating oncogenic transformation of cells (Katayama *et al*, 2004). It also plays a key role in G2/M-phase progression. Studies demonstrated that STK15 also provides drug resistance, and inhibiting its expression by RNA interference can result in potent sensitisation to the chemotherapeutic Taxol in human cancer cells (Hata *et al*, 2005). Our proteomic screen showed a marked upregulation of this kinase in metastatic prostate tumours.

Another protein, MSH2, is encoded for by a gene that is a member of a family of genes long implicated in oncogenesis, and specifically hereditary and sporadic colorectal carcinomas. The gene functions in mismatch recognition during the repair of errors occurring during DNA replication. Identified germ-line missense mutations are known to contribute to the onset of HNPCC, and the gene was shown to be upregulated in primary prostate cancer (Velasco *et al*, 2002), a result concordant with its protein expression in our screen. The same study also demonstrated that MSH2 expression in prostate carcinoma may be a useful prognostic marker for outcome in men with clinically organ-confined prostate carcinoma.

Finally, BM28, whose alias is MCM2, is a well-characterised mini-chromosome maintenance protein, and is known to play an essential role in initiation and regulation of eukaryotic DNA replication. It has been shown to be dysregulated in malignant prostate glands (Meng *et al*, 2001). The same study also suggested that BM28 expression is an independent predictor of disease-free survival after definitive local therapy, and has potential as a molecular marker for clinical outcome in prostate cancer.

These five either established or putative proteomic alterations in prostate cancer only partially reflect a true diversity of function that reaffirms its molecular heterogeneity. Nonetheless, much remains to be performed both experimentally and bioinformatically for these and many more in pathway enrichment, identifying functional targets, regulatory relationships and protein–protein interaction sub-networks to properly contextualise these entities and understand their contribution to prostate cancer progression.

## PROVIDING FUNCTIONAL CONTEXT TO ALTERATIONS

The molecular profiling of prostate cancer tissue for proteomic alterations has certainly advanced the list of targets potentially mediating the neoplastic phenotype and aggressive subtypes. However, systematic biological contextualisation has lagged their initial discovery. This might be expected as a result of labour-intensive experimental characterisation for single proteins, but this also requires mining and computational approaches that can better filter these compendiums, annotated and enriched with their corollary data types, much like those developed for the gene expression domain.

An effective repository and analytical platform providing *meta-analysis* of proteomic alterations in patient material paralleling the successful efforts in cancer microarray analysis would greatly benefit the cancer proteomics community (Rhodes *et al*, 2004). The task of useful integration and compendium development is complicated by the dimensionality and diversity of proteomic data as previously discussed, which is a more tractable problem in the DNA microarray domain. Despite distinct experimental platforms, DNA microarrays generate uniformly single-dimensional, semiquantitative molecular abundance data. However, proteomic data are decidedly higher dimensional, whose diversity of data type includes quantitative, nominal, categorical, binary and more. Nevertheless, the benefits of such a compendium-based meta-analysis at the protein level, a platform that systematises and integrates diverse protein data from the public domain, are many-fold. They include functional enrichment for biological processes, molecular function, and pathway membership. Another is mapping to existing protein–protein interaction data, and integration for targets of existing therapeutics and small-molecule inhibitor libraries to suggest altered combinatorial therapies at the protein level. Others include integration with antibody repositories, and of course with gene expression data, helping the community better understand the regulatory processes mediating the relative discordance witnessed between the two observed in small scale in previous studies.

We foresee such a platform addressing a variety of current complications in extracting truly causal proteomic alterations from larger, noisy data sets. We briefly identify several problems that would be aided by such an analytical approach. First, and particularly problematic, are those stochastic events that are consequences of the neoplastic phenotype as opposed to determinants of it. Filtering for putative alterations in prostate cancer pathogenesis may begin with altered protein expression, but much like at the transcriptome level, this must be qualified and validated.

A second problem is that of tissue specificity, sample selection and annotation. Certainly one of the primary considerations in this context is how to deal with protein markers having tissue specificity. This trait is indicative of the physiological site of metastatic sample analysed, and is indicative only of the latest stages of secondary-site micro- and macro-metastases and perhaps not causal in progression to metastatic disease. Better bioinformatic tools are required to either properly classify these or subtract them from a causal compendium. Such an analytical platform for comparative analysis across large proteomic data sets culled from a variety of distal sites to the primary site of disease will allow for easier assessment of tissue-specific proteomic noise.

A third important distinction to be made is between those protein markers having utility in a prognostic signature and those with actual biological relevance to the disease state, as these are certainly not mutually inclusive. Correlations between entities, be they genes in a transcriptomic signature, or proteins in proteomic ensemble, may drive the value of a given prognostic signature and *a priori* outcome determination, but do not equate to fundamental biological interactions, whether regulatory, binding, kinetic or otherwise. These artefacts are often the consequence of a variety of statistical learning implementations that range from ‘black box’, which masks the structure of the data, to the relatively transparent from which rules can be read with relative ease. However, discriminating between statistical *vs* biological significance remains a fundamental challenge even with the increasing complexity and sophistication of these analytical approaches.

Many of these complications in generating biological context for proteomic alterations implicated in prostate cancer progression are being addressed through smaller-scale integrative approaches that correlate transcriptomic and proteomic experimental data, mine publicly available data sets, and add dimensionality through the inclusion of molecular interaction data. Integrative biology of this type is paradigmatic of a movement towards a systems biology framework that was previously unavailable to biomedical research (Hood *et al*, 2004). We foresee that such a compendium for widespread analysis will facilitate these types of analyses on a far larger scale.

## CONCLUSION

**Figure 2:**
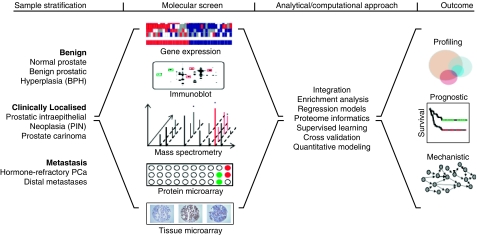
Overview of the conceptual flow in characterising proteomic alterations between prostate cancer stages. Starting from a diverse sample population, a vast array of sample preparation methods and high-throughput technologies can be leveraged to differentially profile tissue stratified by disease progression. A variety of resulting data types, formats, and dimensionality require significant integration and bioinformatic analyses to tease causal entities from the molecular noise and agglomerate the results into one of many desired outcome models motivated by study design.

As technologies and analytical approaches to prostate cancer profiling mature, so will the compendium of proteomic alterations between stages of disease. However, the contextualisation of these molecular determinants in the biology of cancer progression is fundamental to our understanding of their role in the acquisition of phenotypic hallmarks of individual disease. While complex in implementation and variable in capacity for different sample types, high-throughput technologies being used today are key to this effort. Platforms include mass spectrometry and its corollary sample reduction methods, high-throughput immunoblots, tissue microarrays, and protein microarrays. The latter includes reverse-phase implementations, antibody arrays, phage display or others in profiling of serum, amplified immune response and others (Figure 2). This broad sampling of platforms is aiding the characterisation of causative alterations at the protein level that are otherwise masked by the molecular noise or lost to physiological variability. However, these are only tools, and their utility must be informed by a comprehensive understanding of the heterogeneous nature of prostate cancer. In larger profiling studies, end-stage metastatic prostate cancer is often aggregated as a single class of disease; however, diverse metastases demonstrate significant heterogeneity, distal organ specificity, and at the molecular level, equate to distinct subtypes of hormone-refractory prostate cancer (Shah *et al*, 2004). An appreciation for this composition of metastatic disease must filter down to the study of proteomic alterations, not simply stratified by progression through discrete disease stages, but also within-stage subtypes. This approach promises an alternative to end-point analysis, rather a dynamic proteomic model of progression to metastatic prostate cancer, a combinatorial ensemble suitable for efforts ranging from quantitative modelling to therapeutic perturbation.

## Figures and Tables

**Table 1:**
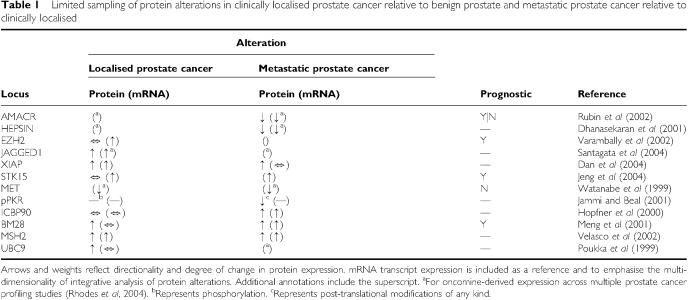
Limited sampling of protein alterations in clinically localised prostate cancer relative to benign prostate and metastatic prostate cancer relative to clinically localised
